# The Treatment of Venereal Diseases in Charity Hospital

**Published:** 1891-04

**Authors:** C. D. Roy

**Affiliations:** House Surgeon Charity Hospital


### Southern Medical Record.

A MONHLY JOURNAL OF MEDICINE AND SURGERY.

VOL. XXI. ATLANTA, GA., APRIL, 1891. No. 4.

### Original Articles

### THE TREATMENT OF VENEREAL DISEASES IN CHARITY HOSPITAL.

BY C. D. ROY, A. B., M. D.,
[House Surgeon Charity Hospital.]

Venereal diseases form a large proportion of the cases which yearly come under the care of a young physician, and the successful treatment of the same is an object much to be aimed at. Especially is the above statement true in large cities like New York. At present there is no hospital in the city which treats exclusively diseases of the genito-urinary organs, although there is a movement now on foot for the establishment of such an institution. Headed by such men as Otis, Taylor, Keyes and others, the realization of the plan should not be a thing of the dim future. While the majority of patients afflicted with venereal diseases are able to be treated at the numerous dispensaries, and at the same time attend to their necessary work, still, there are cases which require a bed-side care, and makes the entrance into a hospital a matter of necessity. Yet there are others so indolent and debauched that they seize with avidity the first chance which offers itself for securing to them a night's lodging. Patients suffering with the class of diseases referred to are not retained in any of the public hospitals except Charity, unless, per chance, it be one of emergency. In this hospital all phases of venereal diseases may be studied, for it is here that the bulk of this class of diseases come, especially from the lower class of the inhabitants. My experience in this institution having now been somewhat extended, I thought that it would be of interest to some to know the various treatment here used in the hospital for the commoner varieties of venereal diseases. If there is any place where the advantages and disadvantages of a remedy, and the method of using it, may be studied with success, it is in a large hospital like this. While the methods of treatment are various, I shall exclude from this article all except those which have been found to be the best. I shall also take in order the treatment of those diseases which I have found to be the most frequent, although the question of frequency is very variable, "changing with the seasons." Chancroids would perhaps hold first rank in order of frequency, and I shall speak of their treatment in the following division:

1. Simple chancroid.
2. Serpiginous chancroid.
3. Complications (a) bubo, (b) phimosis.

My own experience agrees with that of Dr. R. W. Taylor, that in the simple form of chancroids, those presenting a healthy surface, unirritated and with no tendency to spread, the best plan is to use thorough cleanliness, with the application of some mild antiseptic powder, as iodoform. Around this is wrapped bichloride gauze (1-1000) of one-half an inch in width, which is to be changed every day. Instead of iodoform, which is very disagreeable in private practice, you may use the aristol powder, which is very efficient and at the same time almost odorless. This latter powder is gradually superseding the use of iodoform in all cases, as it is a much more elegant preparation to use, and at the same time just as effective. By this daily dressing, the sore will in a few days present the happiest result. Should you have a case of the second variety, or the first develope into the second the course to be pursued is somewhat different. If the sore begins to spread, looks angry or secretes much pus, then some form of cauterization must be adopted. What we use in the hospital is the pure carbolic acid (R. S. P.), and in some instances chromic acid, both of which are excellent, my preference being for the former. The best method of application is to take a wooden tooth-pick, and, having invaginated one end with cotton, to dip this into the acid and apply it to the sore until the whole surface looks blanched or whitened. Over this your iodoform or aristol is sprinkled, the penis wrapped with bichloride gauze, and this dressing left until the second day, when the sore may again be inspected. If the healing process seems to have started, the treatment, as in the first variety, may then be continued. I have sometimes found it advantageous to spread carbolized vaseline (2 grains to ℥) around the sore in order that the gauze may not become adherent by the action of the exudate.

The treatment of buboes I shall not discuss at length, since in a former article in THE RECORD, I described our method of treatment. Since that article another plan has been adopted, known as the "excision method," which bids fair to prove a very successful mode of procedure, but one which I am afraid will not meet the approbation of your patients, since they have such a dreaded idea of cutting. The method is only applicable in the first stage, or the stage of enlargement and irritability of the inguinal glands. If seen at this stage, the patient is etherized, the parts shaved and thoroughly washed with bichloride solution (1-2000), after which you proceed as follows: With a scalpel an incision is made over the swollen gland or glands, usually one, parallel to Poupart's ligament, and with the butt end of your instrument you dissect out the gland, which is usually superficial. Having removed all enlarged glands, the wound is thoroughly irrigated with a solution of bichloride (1-3000), after which the wound is closed with silk sutures. If no suppuration has taken place, the operation will usually be rewarded with the remains of a linear scar, and the disappearance of the bubo symptoms. Many physicians say that poultices should be relegated to practices of the past, as they are of no use, and only favor suppuration. Strange to say, my own experience is different. Several times have I seen the fiercest looking buboes go down and disappear under the continuous application of warm flaxseed poultices. If the first method I mentioned is not adopted, I have found the best results by the continuous use of the poultice referred to, for if it does not eradicate the symptoms, it will soften the tissues and lessen the interval for cutting, which are both points of benefit to the patient, besides the anodyne effect of the moist heat. When suppuration has occurred, the bubo must be opened, and the method of so doing I have described in a former article.

Phimosis complicating chancroids is a frequent occurrence here in the hospital, and much more frequent than text-books would lead one to imagine. It often seems a pity that the Jewish custom is not universally prevalent among all nationalities. In an elongated prepuce the chancroidal virus in some peculiar way often takes effect just behind the corona glandis, or the mucous membrane of the prepuce over this point, and the patient comes in with his whole penis red, swollen and oedematous, with a dirty discharge from the small preputial orifice. When such a case enters the ward, he is ordered to soak his penis for five minutes at a time in hot carbolic acid (two and a-half per cent.) solution, and this he does four or five times during the day. If, after twenty-four hours of such treatment, there is no decline of the symptoms, the prepuce is opened up and the sores inspected. Of course if there are symptoms of strangulation on entrance, the incisions are made immediately. The manner of slitting the prepuce is an important one, and we now use but one method in the hospital. Formerly one incision was made in the median line in front, and the parts inspected as best they could be. Dr. Taylor found the inefficiency of this procedure, and instituted another, which consists in making two lateral incisions just back far enough to leave a margin for future circumcision. By this plan both lips can be turned back and a thorough inspection procured. The sores are then treated daily, the parts being kept thoroughly cleansed and the whole extremity being invested with bichloride gauze. After all the sores have healed, and the inflammation subsided, the flaps are removed, leaving a neat, clean-cut circumcision.

Although syphilis is very manifold in its manifestations, its treatment is very uniform, as is recognized by all authorities. Mercury and the iodides are our sheet-anchor. The initial lesion is generally so mild in its symptoms that, unless it has been irritated, the patient seldom thinks of treatment unless he be a neurotic individual. The blandest treatment is usually the most successful, and nothing acts better than calomel or aristol dusted over the abraded surface. A covering of bichloride gauze is placed over it in order that foreign substances may not produce irritation. As a rule, internal administration is not instituted until there are some of the secondary manifestations. As soon as the latter make their appearance, the patient is put upon the following mixture:

℞ Ferri et quininae cit., ʒ ii.
Hydrarg. protoiodidi, grs. v.
Ext. hyoscyami, grs. iv.

M. Ft. xxx. pills.

One of these is given three times daily and increased one every other day until some mercurial symptoms are manifested. For mercurial stomatitis, fifteen grains of chlorate of potash is administered internally, and the mouth washed with a weak solution of tincture of iodide and myrrh. The administration of the iodide of potash alternately with mercury is often beneficial. In late secondary or early tertiary symptoms, the "mixed treatment" is very efficacious:

℞ Hydrarg. biniodidi, gr. ½.

Potash iodidi, ʒ ii.

Syrp. sarsaparil. Co.

Aquae, aa. ℥ i.

Dissolve and mix. Sig. One teaspoonful three times daily.

In the tertiary forms the iodides still remain first. They are administered in increasing doses until the symptoms of iodism are produced, when it is again reduced. The anaemic state of the blood is always considered, and iron is administered when necessary. If the internal use of mercury disagrees with the patient, the inunctions are often substituted with good results.

Venereal warts, or condylomata, is an accompaniment frequently met with, especially around the genitals of the female. For these I might almost say there was a specific:

℞ Hydrarg. bichlo., ʒi.

Collodii flex., ℥i.

M. With this solution the condylomatas are thoroughly painted, and after about two or three applications the excrescences drop off, leaving a clean, smooth surface.

*Gonorrhœa.*—In a charity hospital like this, it is very seldom that patients come in with a gonorrhœa in the acute stage. By acuteness I mean the very commencement of the discharge. They generally come in after the discharge has lasted a week or more. If the history gives any acute character at all, and not that of chronicity, the patient is put upon the following mixture:

℞ Potassi acet., ℥i.
Aquae, ℥iv.

M. Sig. One teaspoonful three times a day. At the same time the urethra is irrigated by hot bichloride solution (1-30000) three times daily. No stimulating drinks in the way of tea or coffee are allowed. If under this regime the symptoms do not abate, mild injections of the following solution are instituted:

℞ Zinci sulph., grs. ii.
Aquae, ℥i. M.

The urine is always kept bland and unirritating, and I have found the above method very satisfactory.

In giving these modes of treatment, it must be borne in mind that I am giving those which have proven most efficacious among the large number which is yearly treated in the hospital. Gleet, dependent upon strictures, I will not mention, lest this article should be too prolix. Among the complications, or rather results of gonorrhoea, are epidydimitis and orchitis, the former being much the more frequent. For a while the plan of treatment consisted in painting the inflamed testicle with a strong solution of nitrate of silver during the height of the attack. At present the treatment differs. During the height of the inflammatory attack a tobacco poultice is made to surround the affected parts, and kept on until the acute stage begins to subside. The poultice is made according to the formula given by Dr. Keyes in his excellent work, from which I quote: "Mix an ounce of fine cut tobacco in ten ounces of hot water, bringing the whole to a boil while stirring it briskly, and then add ground flaxseed, with or without ground elm bark, until the proper consistency of a poultice is obtained. This mass is surrounded with fine muslin and the poultice applied. The poultice should not be allowed to become cool. After the acute stage is passed a solution of nitrate of silver (40 grains to ℥) is applied with a brush, and the testicle supported with straps. At no period should the scrotum be allowed to hang down. This latter application, though painful at first, acts as an anodyne, and hastens restoration.

These practical points have been given in hopes that they may prove of service to some whose experience is yet limited on this line. I have not attempted to add any new ideas in regard to the pathology or morbid anatomy of any of the diseases herein contained, but have simply given those points as being practical in their bearing.

